# Across the COVID-19 Waves; Assessing Temporal Fluctuations in Perceived Stress, Post-Traumatic Symptoms, Worry, Anxiety and Civic Moral Disengagement over One Year of Pandemic

**DOI:** 10.3390/ijerph18115651

**Published:** 2021-05-25

**Authors:** Alessio Gori, Eleonora Topino

**Affiliations:** 1 Department of Health Sciences, University of Florence, Via di San Salvi 12, pad. 26, 50135 Firenze, Italy; 2 Department of Human Sciences, LUMSA University of Rome, Via della Traspontina 21, 00193 Rome, Italy; eleonora.topino@gmail.com

**Keywords:** COVID-19 pandemic, trend study, mental health, perceived stress, post-traumatic symptoms, worry, anxiety, civic moral disengagement

## Abstract

This study aimed at investigating the psychological effect of the COVID-19 pandemic in Italy by analysing the trends of perceived stress, post-traumatic symptoms, state anxiety, worry, and civic moral disengagement in four different moments from March 2020 to March 2021. The study involved a total of 1827 Italian participants (30% men and 70% women; *M_age_* = 34.72; *SD* = 12.40) divided into four groups to which an online survey was administered. The first group completed the survey in March 2020, the second one in August 2020, the third one in November 2020, and the fourth one in March 2021. Results highlighted significant decreases in post-traumatic symptoms and a significant increase in civic moral disengagement over the first year of the COVID-19 pandemic. The levels of perceived stress, worry, and state anxiety remained constant. The correlations between the variables at different times were also explored, as well as gender differences over the year. The COVID-19 emergency has had significant effects on the mental state of the population, with important repercussions for individual and collective well-being during but probably also after the pandemic. This study offers a clear snapshot of the psychological outcomes over one COVID-19 pandemic year, providing important information that may contribute to tailor more effective interventions for mental health.

## 1. Introduction

The COVID-19 disease is caused by the severe acute respiratory syndrome coronavirus 2 (SARS-CoV-2), and it has brought about sudden, drastic, and unexpected changes in daily life for people around the world [1]. The first cases appeared in Wuhan, China, in late December 2019; from there, the virus then quickly spread around the world to the point that the World Health Organization (WHO) classified the COVID-19 emergency as a global pandemic on 11 March 2020 [2]. Italy was the first European country with a diagnosed case of COVID-19 on 20 February 2020. This resulted in a succession of government measures aimed at limiting the spread of the virus, including a national lockdown which lasted almost two months, from 10 March to 3 May 2020. From 4 May 2020, phases of localized closures and gradual re-openings of financial and commercial activities were then alternated [3]. As in Italy, other countries also adopted numerous preventive and virus-containment measures over time [4]. Given this framework, the international scientific literature highlights how the pandemic has had an impact not only on the sphere of physical health but how it has also caused psychological disorders (see Vindegaard and Benros [5] for a review). Restrictions to contain the virus, economic instability, risk of contagion, uncertainty about the future, unpredictability of the virus spread, and the growing emergence of its variants may cause significant and lasting effects on mental health [6]. Some cross-sectional studies have already shown the emotional impact of the lockdown and the early stages of the pandemic, highlighting high levels of distress [7,8], post-traumatic symptoms [7,9], worry [10,11], and anxiety [7,11], underlining the strong link between compliance with restrictions and moral disengagement [12], and noting the differences in the effects on mental health according to gender [13,14]. One year after the outbreak, with the in-place vaccination campaign, maintenance of preventive measures, and alternation of localized closure phases, it could be important to monitor the progress of the psychological effects of the health emergency in order to promote adequate and targeted implementation of supportive and therapeutic interventions [15]. To the authors’ knowledge, some longitudinal studies have already analyzed the consequences for mental health due to the COVID-19 pandemic but covering only the first weeks or months of the outbreak (e.g., the ones of Okruszek and colleagues [16], Hyland and colleagues [17], Pierce and colleagues [18], and Wang and colleagues [19]). At the time of writing, there is still a lack of research showing trajectories of psychological dimensions in the general population across longer periods. To fill this gap, this study questions the constancy of some psychological dimensions during the health emergency, adopting an exploratory approach. Therefore, this research aims at gaining a snapshot of mental health and some psychological outcomes over the first year of the COVID-19 pandemic in Italy by investigating the trends of perceived stress, post-traumatic symptoms, worry, state anxiety, and civic moral disengagement in four different moments from March 2020 to March 2021.

## 2. Materials and Methods

### 2.1. Participants and Procedure

The study involved 1827 Italian participants living in Italy (30% men and 70% women) with an age range of 18 to 85 years (*M_age_* = 34.72; *SD* = 12.40). They were recruited in four different moments during the COVID-19 pandemic: 814 individuals (32% men and 68% women; *M_age_* = 35.85; *SD* = 12.38) were involved in March 2020, 523 individuals (26% men and 74% women; *M_age_* = 32.87; *SD* = 10.55) were involved in August 2020, 250 individuals (39% men and 61% women; *M_age_* = 36.57; *SD* = 14.57) were involved in November 2020, and 240 individuals (23% men and 77% women; *M_age_* = 33.00; *SD* = 13.80) were involved in March 2021. The administrations were carried out on the Internet with an online survey by using the Google Forms platform after sending out an anonymous link with a snowball sampling method. All participants were informed of the objectives of the study and provided informed consent electronically before starting. Each respondent answered the survey only one time, as required by the instructions. Procedures were carried out according to current ethical guidelines and were approved by the Ethical Committee of the Integrated Psychodynamic Psychotherapy Institute (IPPI; ethical approval number 001/2020).

### 2.2. Measures

#### 2.2.1. Ten-Item Perceived Stress Scale (PSS−10) 

The Ten-Item Perceived Stress Scale (PSS−10) is a self-report measure for the assessment of the level of stress experienced in the respondents that asks them for their feelings and thoughts during the last month [20]. It consists of 10 items scored on a five-point Likert scale, from 0 (never) to 4 (very often). The total score (obtained by summing all the items) of the Italian translation used in the present study was developed by Fossati [21], and it showed satisfactory internal consistency with a Cronbach’s α of 0.87 in the present sample.

#### 2.2.2. Impact of Event Scale—Revised (I-IES-R)

The Impact of Event Scale—Revised (I-IES-R) is a self-report measure for the assessment of post-traumatic distress [22]. It consists of 22 items scored on a five-point Likert scale, from 0 (not at all) to 4 (extremely), grouped into three factors: intrusion (consisting of the sum of eight items); avoidance (consisting of the sum of eight items); and hyperarousal (consisting of the sum of six items). Furthermore, a total score of impact of event could be obtained by summing all the items. In this study, the Italian version developed by Craparo and colleagues [23] was used, which showed an excellent internal consistency (α = 0.93).

#### 2.2.3. State-Trait Anxiety Inventory—Form X3 (STAI—X3)

The State-Trait Anxiety Inventory [24] is a self-report measure for the assessment of the level of anxiety. In this study, the short Italian version Form X3 of Vidotto and Bertolotti [25] was used. It consists of 10 items evaluating state anxiety on a four-point Likert scale, from 1 (not at all) to 4 (very much so). In the present sample, the total score (obtained by summing all the items) showed excellent internal consistency with a Cronbach’s α of 0.93. 

#### 2.2.4. Penn State Worry Questionnaire (PSWQ)

The Penn State Worry Questionnaire (PSWQ) is a self-report for the assessment of pervasive and uncontrollable worry [26]. It consists of 16 items rated on a five-point Likert scale from 1 (not at all typical) to 5 (very typical). The total score (obtained by summing all the items) of the Italian version of Meloni and Grana [27] was used for this study, showing to have excellent reliability in the present sample (α = 0.93). 

#### 2.2.5. Civic Moral Disengagement Scale (CMDs)

The Civic Moral Disengagement Scale (CMDs) is a self-report measure for the assessment of the civic moral disengagement mechanisms by which moral self-sanctions can be disengaged from aggressive conduct: moral justification, euphemistic language, advantageous comparison, displacement of responsibility, diffusion of responsibility, distorting consequences, attribution of blame, and dehumanization [28]. The scale consists of 32 items scored on a five-point Likert scale ranging from 1 (agree not at all) to 5 (completely agree). It obtains the 8 subscales concerning the different civic moral disengagement mechanisms (each obtained by summing the 4 corresponding items) and a total score (obtained by summing all the items). The CMDs showed excellent psychometric properties with a Cronbach’s α of 0.90 for the total score in the present sample.

### 2.3. Data Analysis

All the analyses were performed with the SPSS software (IBM-SPSS 25.0 version, IBM, Armonk, NY, USA) for Windows. Pearson’s correlations were used to investigate the association between the variables in four different moments (T1: March 2020; T2: August 2020; T3: November 2020; T4: March 2021). The trends of perceived stress, post-traumatic symptoms and its subscales, worry, state anxiety, as well as civic moral disengagement and its subscales during the first year of the COVID-19 pandemic were explored by carrying out a two-way multivariate analysis of variance (MANOVA). The differences in the mean scores of the psychological variables (dependent variables) between four different times (T1, T2, T3, T4) and considering the effect of gender (independent variables), were assessed. Separate analyses of variance (ANOVAs) were conducted as follow-up tests by using a Bonferroni-adjusted *p*-value of 0.0125 as criterion of significance. Post hoc analyses using a Scheffé test were implemented to support the interpretation of the differences in mean scores where needed. Except for the ANOVAs, significance was set at *p* < 0.05.

## 3. Results

Pearson’s correlation showed significant and positive associations among the total scores of the variables over the four analyzed moments with some exceptions. In particular, in March 2020, civic moral disengagement did not correlate with worry (*r* = 0.058, *p* = 0.096), while in August 2020, these two variables were weakly and positively associated (*r* = 0.099, *p* < 0.05; see Table 1). In November 2020, civic moral disengagement did not show significant correlations with the other variables (see Table 2). On the other hand, in March 2021, civic moral disengagement was positively and significant associated with state anxiety (*r* = 0.135, *p* < 0.05) and post-traumatic symptoms (*r* = 0.158, *p* < 0.05) but not with perceived stress (*r* = 0.103, *p* = 0.112) and worry (*r* = 0.090, *p* = 0.162; see Table 2).

The two-way MANOVA showed that the interaction between time and gender did not have a statistically significant effect on the dependent variables: *F*(42, 5358.227) = 0.998, *p* = 0.475; Wilks’ Λ = 0.977. However, there was a statistically significant difference in the psychological outcomes based on time, *F*(42, 5358.227) = 5.927, *p* < 0.001; Wilk’s Λ = 0.874, as well as a statistically significant difference in the psychological outcomes based on gender, *F*(14, 1806) = 22.322, *p* < 0.001; Wilk’s Λ = 0.852. Therefore, the effect of each independent variable on the dependent ones was not influenced by the other.

Concerning time, the separate ANOVAs conducted for each dependent variable showed significant effects on post-traumatic symptoms, *F*(3, 1819) = 18.289, *p* < 0.001, and civic moral disengagement, *F*(3, 1819) = 4.794, *p* < 0.001 (see Table 3). Specifically, Scheffé’s post hoc analysis indicated that post-traumatic symptoms were significantly higher in March 2020 (T1) than in August 2020 (T2), November 2020 (T3), and March 2021 (T4). More in depth, significant decreases between March 2020 (T1) and the other times were found also on the subdimensions of post-traumatic symptoms: intrusion, avoidance, and hyperarousal (see Table 3). Concerning civic moral disengagement, the scores were significantly higher in November 2021 than in that of March 2020. More in depth, significant increases were found also on some subscales of civic moral disengagement over the course of the four moments: euphemistic language, advantageous comparison, diffusion of responsibility, distorting consequences, and attribution of blame (see Table 3). Finally, there were no significant differences in perceived stress, worry, and state anxiety between the four times (see Table 3).

**Figure 1:**
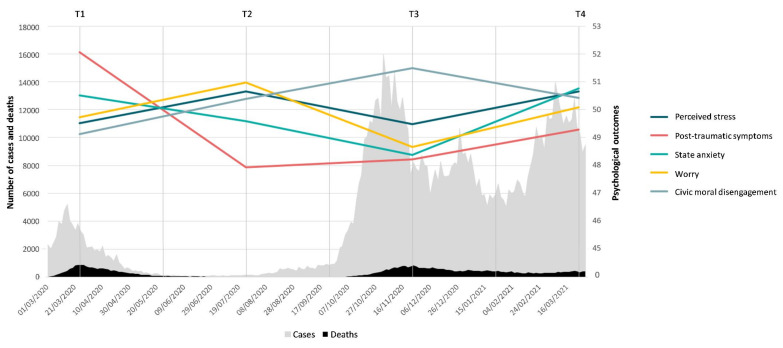
Trend of the perceived stress, post-traumatic symptoms, state anxiety, worry, and civic moral disengagement and cases and deaths during one year of COVID-19. Note: T1, March 2020; T2, August 2020; T3, November 2020; T4, March 2021; Cases = number of confirmed SARS-CoV-2 virus infections; Deaths = number of cases that have died. The data for number of cases and deaths were from the report of “Epicentro” [29]; when the number of deaths was < 5, it was approximated to zero in creating the chart. For the psychological outcomes, raw data were converted into *t*-scores to get better visibility in the chart.

In Figure 1, the trend of the psychological outcomes in four different moments over the first year of the COVID-19 pandemic are shown together with the number of cases and deaths in Italy [29].

Concerning gender, the separate ANOVAs conducted for each dependent variable showed that women had significantly higher scores than men in perceived stress, *F*(1, 1819) = 51.996, *p* < 0.001; worry, *F*(1, 1819) = 54.700, *p* < 0.029; state anxiety, *F*(1, 1819) = 33.814, *p* < 0.001; and post-traumatic symptoms, *F*(1, 1819) = 36.579, *p* < 0.001 (see Table 4). 

Finally, concerning civic moral disengagement, significantly higher values were found in men than in women: (*F*(1, 1819) = 97.746, *p* < 0.001 (see Table 4).

## 4. Discussion

The pandemic has strongly impacted people’s well-being, as shown by some studies that have found the onset of relevant emotional outcomes by comparing the state of the participants before and during the COVID-19 emergency (e.g., Pierce and colleagues [18]). To expand such evidence, the present trend study describes the psychological effects this situation has had on the general population in Italy over the first year of pandemic to track the trajectories of dimensions that could influence people’s mental health.

### 4.1. Associations among the Psychological Outcomes over Time

The analyses of the correlation between the variables over the first year of the pandemic highlighted continuity in relation between post-traumatic symptoms, state anxiety, worry, and perceived stress. On the other hand, civic moral disengagement showed greater discontinuity from this point of view. Although in March 2020, August 2020, and March 2021, it showed significant relationships with state anxiety and post-traumatic stress, as well as sometimes with worry (August 2020) and perceived stress (March 2020 and August 2020), no significant associations were found in November 2020, when the highest CMDs scores were recorded (see Table 3). Since it was also associated to intolerance of uncertainty in previous research [12], these data could indicate that civic moral disengagement may be seen as a mechanism activated in association with a state of fatigue, uncertainty, and discomfort due to new peaks of contagion (see Figure 1) [29] and the consequent restrictions and limitations imposed in that phase. However, it did not appear to be in itself sufficient to contrast the levels of malaise given that no negative relationship with the other psychological outcomes were found in its peak phase.

### 4.2. Trends of the Psychological Outcomes Over Time

Delving deeper into the trends of the psychological outcomes during the first year of the COVID-19 pandemic, results highlighted significant variations in post-traumatic symptoms and civic moral disengagement. On the other hand, the levels of perceived stress, worry, and state anxiety remained constant. 

In more detail, post-traumatic symptoms significantly declined after the first phase in March 2020. Indeed, post-traumatic stress can be read as a response to a perception of danger that derives from a sudden situation and the consequent profound changes related to it. Consistently, the sub-dimensions of intrusion, avoidance, and hyperarousal were also reported significantly more in March 2020, after an initial peak of infections (see Figure 1) [29] and during the first and consequent Italian lockdown, than in the subsequent phases. A reduction in these symptoms over the first year of pandemic may be linked to a relaxation of the restrictive measures, for which previous studies have highlighted a significant psychological impact [30]: indeed, the highest scores were recorded in an initial phase of limitations. Furthermore, this expands the preliminary longitudinal study of Wang and colleagues [18] that identified a statistically significant decrease in post-traumatic symptoms after four weeks (from 3 January to 1 March 2020) in Chinese participants.

Although the results showed decreasing trends in post-traumatic symptoms, no significant variations in the levels of perceived stress, worry, and state anxiety were found. 

Such findings appear relevant and indicated a prolonged state of stress generated by the pandemic, suggesting the risk of chronicization, with consequent repercussions for people’s health [31]. This may be read considering the extent of COVID-19 related issues, which, in addition to being a health crisis [32] also has significant social and economic consequences [33,34,35,36]. The pandemic, in fact, is a source of great financial insecurity, sometimes causing job loss and changes in the working modalities (e.g., concerning workplace, working hours, ways of communicating with colleagues and superiors), which can lead to increased stress and have a significant impact on psychological wellbeing (see Kniffin and colleagues [36] for a review). These results further enrich the ones of Wang and colleagues [19], which reported no significant longitudinal changes in stress in Chinese participants (from 3 January to 1 March 2020).

Consistently, the findings also suggested persistent levels of worry related to the pandemic. Indeed, the characteristics of the disease (perceived as invisible and intangible); the uncertainty about the possibility, timing, and modalities of healing of patients; as well as the imposed change of habits for the protection of public health can be a source of sense of fear and concern about the dangerousness of COVID-19, of being personally infected, or that someone close could be infected [37,38,39]. Therefore, this can translate into a lasting perception of not feeling safe, which remains constant even in the phases of reduction of cases. These results are consistent with those obtained in the longitudinal study of Okruszek and colleagues [16], which highlighted a continuity in COVID-19 risk perception in Poland participants over about two weeks (from 15 March to 31 March 2020).

Furthermore, in line with previous studies that explain the association between fear and anxiety at the time of COVID-19 [11], persistent levels of state anxiety were found. Anxiety derives from the anticipation of a real or felt threat [40] and may be associated with a state of insecurity that accompanies situations perceived as unpredictable and uncontrollable (see Coelho and colleagues [41] for a review). Such findings could therefore be a manifestation of a constant sense of uncertainty of the future due to the course of the pandemic, which had progressive peaks in the number of infections across the year [29], as well as to the further consequences and challenges at different levels that could ensue [42,43,44]. Furthermore, these results integrate the preliminary longitudinal study of Hyland and colleagues [17], which found no change in the prevalence of generalized anxiety disorder across during six weeks of lockdown (from 31 March–5 April, to 30 April–14 May 2020) in adults in the Republic of Ireland. 

Finally, a significant increase civic moral disengagement was highlighted, suggesting a higher use of cognitive mechanisms aimed to disengage moral self-sanctions due to civic reprehensible conduct. More specifically, already in August 2020, a tendency to label own actions with euphemistic language emerged, but the greatest number of mechanisms were found in November 2020: compared to March 2020, people showed an increase in the tendency to spread responsibility to the community, thus making the individual contribution indistinguishable (diffusion of responsibility), as well as in the avoidance in recognizing one’s own blameworthy behavior by ignoring or distorting its consequences (distorting consequences) and, in the parallel, attribution of blame towards others. Furthermore, higher tendencies of distorting consequences and comparing own behavior with worse ones (advantageous comparison) were found in March 2021 than in the first phases of the pandemic (March 2020). Since higher levels of moral disengagement have been associated with lower conformity to some rules and restrictions aimed at protecting physical health during the COVID-19 emergency [12], such findings appear particularly relevant. These mechanisms, in fact, allow the avoidance of assuming full responsibility for one’s actions while being able to recognize one’s moral obligations and distinguish what is right from what is wrong [45]; it is clear, therefore, how this can have high social and personal costs in times of a pandemic given the importance of adhering to preventive measures to manage the virus spread.

### 4.3. Gender Differences in the Psychological Outcomes Over Time

Significant differences between men and women were identified in the psychological outcomes, which proved to be constant over the first year of the COVID-19 pandemic: the relationships between gender and the observed variable did not change based on time. Results highlighted significant higher scores in civic moral disengagement in men than in women, which was consistent with previous research [28]; this indicated that, also during the pandemic, males are more inclined than females to disengage morally from their conduct. Regarding the other psychological outcomes, women showed higher scores than men in perceived stress, worry, state anxiety, and post-traumatic symptoms, expanding and giving continuity to the results of previous studies [14,46,47,48]. Indeed, the pandemic seems to impact men and women differently, since the former have increased time to spend on family care and tend to change their working hours during lockdown, and this has been associated with psychological discomfort [48]. Furthermore, the continuity over time in differences showed in this study was consistent with previous surveys in other settings regarding health crises (e.g., radiation in Fukushima after the 2011 nuclear reactor meltdown), which have found significant gender differences in health risks assessment, with women more likely to remain more concerned than men over the long term [49]. 

### 4.4. Strengths and Limitations of This Study

This study has several limitations that need to be highlighted to favor an appropriate interpretation of the results. First, data were collected via digital means with a snowball sampling by spreading an anonymous survey online. This implies that those who did not have Internet access may be underrepresented. Therefore, future research could use a more inclusive recruitment procedure to avoid the underestimation of psychological outcomes in specific categories of population. Furthermore, the samples are not homogeneous from a numerical point of view, and the participants were different in each phase of administration. The trend study design (also called independent longitudinal design) only allows a snapshot of changes at the social level. However, it might be useful and extremely informative to acquire data on the same participants over the course of this time period, testing them several times. Therefore, an important challenge for future research is setting up longitudinal studies in which the same respondents are evaluated at different moments. Moreover, no data were collected regarding the different ethnic groups living in Italy nor information regarding the socio-economic status or the provenience region of the participants. These could be important aspects to be explored in future research, integrating other preliminary studies that suggest the existence of an unequal socioeconomic gradient (including differences in age, gender, and race between poverty groups) in the demographic and clinical presentation of COVID-19 patients [50], also considering the different distribution of the virus in the Italian country in each phase [27]. Finally, the use of self-report measures inherently introduces measuring biases, especially the social desirability one which may lead to underreporting due to the stigma attached to mental illness. To face this issue, the integration of structured or semi-structured interviews following a multimethod-multimodal approach could be an important suggestion for future research.

Despite these limitations, this research also has valuable strengths. Indeed, this trend study offers clear and important information of the psychological state over the first year of the COVID-19 pandemic. Specifically, the trajectories concerning mental health and civic moral disengagement were highlighted, allowing a better understanding of the emergency psychological outcomes. These may have important repercussions for individual and collective well-being during but also after the pandemic. Therefore, such findings suggest the need to implement effective public mental health interventions supported by the integration of clinical practice and research in order to prevent and limit negative effects of COVID-19 on the mental health of the population.

## 5. Conclusions

In conclusion, detailed monitoring of longer-term psychological outcomes is essential to tailor timely, up-to-date interventions. Therefore, by offering a snapshot concerning the trends of post-traumatic symptoms, perceived stress, sorry, state anxiety, and civic moral disengagement over a year of COVID-19 pandemic, this research allows integration and enrichment of the previous cross-sectional studies concerning the effects of the outbreak on mental health as well as the preliminary longitudinal studies that focused on the first weeks of the emergency. The broader vision offered, therefore, made it possible to analyze the trajectories of some psychological variables in the cyclical succession of closures, containments, relaxation of the preventive measures, and restrictions again that alternated in Italy during the first year of the pandemic. The understanding of the long-term psychological outcomes of the COVID-19 pandemic may give an important key in fostering effective management of mental health consequences both during the emergency and when it is over, also considering gender differences. More specifically, these results highlight the importance of interventions to prevent post-traumatic symptoms by acting early, since they seem to be related to the disorientation of the first phases; favor the development of the ability to contain and cope with stress, worry, and state anxiety, which appeared to be the most persistent elements during the crisis; monitor the increases in civic moral disengagement, which seemed to be related to a state of fatigue and uncertainty and, as demonstrated by previous research [12], can be associated with lower conformity to some rules and restrictions. Therefore, this study may contribute to provide useful data to tailor interventions in clinical practice for both the present health crisis and future emergencies as well as underline the need for future research of further trend analysis of psychological outcomes during the next phases of pandemic.

## Figures and Tables

**Table 1:** Correlation among the variables in March 2020 (T1, over the diagonal) and August 2020 (T2, below the diagonal).

	1	2	3	4	5	6	7	8	9	10	11	12	13	14	15	16
1. PSS	1	0.645 **	0.690 **	0.632 **	0.396 **	0.600 **	0.695 **	0.150 **	0.087 *	0.030	0.093 **	0.089 *	0.152 **	0.112 **	0.125 **	0.158 **
2. PSWQ	0.588 **	1	0.622 **	0.543 **	0.348 **	0.508 **	0.600 **	0.058	0.040	0.002	0.034	0.018	0.082 *	0.025	0.066	0.057
3. STAIX	0.631 **	0.609 **	1	0.578 **	0.343 **	0.561 **	0.640 **	0.145 **	0.111 **	0.024	0.076 *	0.080 *	0.111 **	0.144 **	0.151 **	0.123 **
4. IESR	0.536 **	0.416 **	0.462 **	1	0.835 **	0.935 **	0.894 **	0.119 **	0.028	0.040	0.100 **	0.097 **	0.080 *	0.098 **	0.130 **	0.105 **
5. IESR (F1)	0.389 **	0.273 **	0.312 **	0.861 **	1	0.650 **	0.585 **	0.117 **	0.029	0.050	0.077 *	0.102 **	0.091 **	0.099 **	0.119 **	0.094 **
6. IESR (F2)	0.517 **	0.412 **	0.434 **	0.936 **	0.669 **	1	0.816 **	0.111 **	0.037	0.030	0.095 **	0.099 **	0.062	0.086 *	0.116 **	0.103 **
7. IESR (F3)	0.557 **	0.450 **	0.522 **	0.934 **	0.695 **	0.864 **	1	0.090 *	0.004	0.026	0.095 **	0.053	0.062	0.076 *	0.114 **	0.080 *
8. CMDs	0.109 *	0.099 *	0.152 **	0.106 *	0.144 **	0.086 *	0.058	1	0.717 **	0.648 **	0.712 **	0.701 **	0.750 **	0.696 **	0.754 **	0.684 **
9. CMDs (F1)	0.011	0.016	0.077	0.050	0.085	0.032	0.020	0.761 **	1	0.401 **	0.394 **	0.428 **	0.412 **	0.418 **	0.490 **	0.475 **
10. CMDs (F2)	0.076	0.082	0.082	0.060	0.072	0.055	0.037	0.711 **	0.503 **	1	0.530 **	0.344 **	0.423 **	0.357 **	0.310 **	0.312 **
11. CMDs (F3)	0.165 **	0.164 **	0.221 **	0.115 **	0.091 *	0.106 *	0.117 **	0.766 **	0.449 **	0.591 **	1	0.444 **	0.489 **	0.510 **	0.397 **	0.335 **
12. CMDs (F4)	0.039	0.017	0.105 *	0.043	0.099 *	0.028	−0.012	0.761 **	0.536 **	0.429 **	0.553 **	1	0.515 **	0.461 **	0.465 **	0.358 **
13. CMDs (F5)	0.142 **	0.139 **	0.164 **	0.119 **	0.155 **	0.102 *	0.067	0.721 **	0.466 **	0.428 **	0.504 **	0.516 **	1	0.491 **	0.522 **	0.407 **
14. CMDs (F6)	0.095 *	0.084	0.137 **	0.084	0.087 *	0.077	0.065	0.705 **	0.428 **	0.435 **	0.568 **	0.487 **	0.515 **	1	0.454 **	0.340 **
15. CMDs (F7)	0.086 *	0.048	0.071	0.131 **	0.189 **	0.096 *	0.074	0.746 **	0.519 **	0.387 **	0.462 **	0.500 **	0.458 **	0.444 **	1	0.565 **
16. CMDs (F8)	0.033	0.041	0.053	0.019	0.059	0.013	−0.021	0.683 **	0.526 **	0.347 **	0.387 **	0.450 **	0.392 **	0.324 **	0.539 **	1

Note: * Correlation is significant at the 0.05 level (two-tailed). ** Correlation is significant at the 0.01 level (2-tailed). PSS, Perceived Stress Scale; PSWQ, Penn State Worry Questionnaire; STAIX, State-Trait Anxiety Inventory—Form X3; IESR, Impact of Event Scale—revised; IESR (F1), Intrusion (Impact of Event Scale—revised); IESR (F2), Avoidance (Impact of Event Scale—revised); IESR (F3), Hyperarousal (Impact of Event Scale—revised); CMDs, Civic Moral Disengagement Scale; CMDs (F1), Moral justification (Civic Moral Disengagement Scale); CMDs (F2), Euphemistic language (Civic Moral Disengagement Scale); CMDs (F3), Advantageous comparison (Civic Moral Disengagement Scale); CMDs (F4), Displacement of responsibility (Civic Moral Disengagement Scale); CMDs (F5), Diffusion of responsibility (Civic Moral Disengagement Scale); CMDs (F6), Distorting consequences (Civic Moral Disengagement Scale); CMDs (F7), Attribution of blame (Civic Moral Disengagement Scale); CMDs (F8), Dehumanization (Civic Moral Disengagement Scale).

**Table 2:** Correlation among the variables in November 2020 (T3, over the diagonal) and March 2021 (T4, below the diagonal).

	1	2	3	4	5	6	7	8	9	10	11	12	13	14	15	16
1. PSS	1	0.592 **	0.598 **	0.529 **	0.371 **	0.513 **	0.573 **	0.074	0.110	0.151 *	0.157 *	0.022	−0.073	−0.017	0.049	0.045
2. PSWQ	0.526 **	1	0.480 **	0.476 **	0.354 **	0.474 **	0.476 **	−0.040	−0.017	−0.015	0.057	−0.054	−0.094	−0.055	−0.036	−0.024
3. STAIX	0.643 **	0.447 **	1	0.477 **	0.330 **	0.481 **	0.501 **	0.083	0.088	0.069	0.160 *	0.061	−0.047	0.025	0.077	0.074
4. IESR	0.546 **	0.415 **	0.527 **	1	0.882 **	0.940 **	0.907 **	0.087	0.076	0.135 *	0.108	0.030	0.012	−0.026	0.110	0.066
5. IESR (F1)	0.400 **	0.344 **	0.422 **	0.880 **	1	0.721 **	0.674 **	0.082	0.100	0.107	0.078	0.025	0.009	−0.026	0.113	0.078
6. IESR (F2)	0.538 **	0.386 **	0.481 **	0.946 **	0.732 **	1	0.831 **	0.087	0.058	0.124 *	0.134 *	0.034	0.013	−0.010	0.105	0.053
7. IESR (F3)	0.559 **	0.408 **	0.548 **	0.910 **	0.670 **	0.841 **	1	0.066	0.048	0.141 *	0.076	0.021	0.012	−0.037	0.079	0.050
8. CMDs	0.103	0.090	0.135 *	0.158 *	0.220 **	0.087	0.130 *	1	0.774 **	0.743 **	0.718 **	0.755 **	0.786 **	0.733 **	0.799 **	0.684 **
9. CMDs (F1)	0.076	0.035	0.131 *	0.151 *	0.230 **	0.088	0.096	0.828 **	1	0.577 **	0.564 **	0.468 **	0.448 **	0.483 **	0.587 **	0.513 **
10. CMDs (F2)	0.110	0.118	0.142 *	0.143 *	0.181 **	0.082	0.136 *	0.834 **	0.667 **	1	0.573 **	0.442 **	0.465 **	0.439 **	0.513 **	0.420 **
11. CMDs (F3)	0.081	0.080	0.113	0.139 *	0.227 **	0.061	0.096	0.835 **	0.650 **	0.677 **	1	0.523 **	0.451 **	0.441 **	0.459 **	0.324 **
12. CMDs (F4)	0.100	0.095	0.183 **	0.148 *	0.183 **	0.107	0.118	0.781 **	0.549 **	0.557 **	0.692 **	1	0.658 **	0.573 **	0.507 **	0.404 **
13. CMDs (F5)	0.087	0.050	0.133 *	0.143 *	0.164 *	0.085	0.149 *	0.884 **	0.707 **	0.704 **	0.697 **	0.657 **	1	0.637 **	0.609 **	0.429 **
14. CMDs (F6)	0.103	0.040	0.117	0.143 *	0.183 **	0.083	0.132 *	0.882 **	0.655 **	0.716 **	0.733 **	0.709 **	0.798 **	1	0.458 **	0.394 **
15. CMDs (F7)	0.067	0.088	0.087	0.088	0.139 *	0.026	0.081	0.848 **	0.658 **	0.672 **	0.590 **	0.597 **	0.728 **	0.688 **	1	0.595 **
16. CMDs (F8)	0.060	0.094	−0.005	0.094	0.151 *	0.050	0.058	0.738 **	0.582 **	0.524 **	0.505 **	0.465 **	0.571 **	0.560 **	0.666 **	1

Note: * Correlation is significant at the 0.05 level (two-tailed). ** Correlation is significant at the 0.01 level (two-tailed). PSS, Perceived Stress Scale; PSWQ, Penn State Worry Questionnaire; STAIX, State-Trait Anxiety Inventory—Form X3; IESR, Impact of Event Scale—revised; IESR (F1), Intrusion (Impact of Event Scale—revised); IESR (F2), Avoidance (Impact of Event Scale—revised); IESR (F3), Hyperarousal (Impact of Event Scale—revised); CMDs, Civic Moral Disengagement Scale; CMDs (F1), Moral justification (Civic Moral Disengagement Scale); CMDs (F2), Euphemistic language (Civic Moral Disengagement Scale); CMDs (F3), Advantageous comparison (Civic Moral Disengagement Scale); CMDs (F4), Displacement of responsibility (Civic Moral Disengagement Scale); CMDs (F5), Diffusion of responsibility (Civic Moral Disengagement Scale); CMDs (F6), Distorting consequences (Civic Moral Disengagement Scale); CMDs (F7), Attribution of blame (Civic Moral Disengagement Scale); CMDs (F8), Dehumanization (Civic Moral Disengagement Scale).

**Table 3:** Means, standard deviation, and comparisons of perceived stress, post-traumatic symptoms, state anxiety, worry, and civic moral disengagement between COVID-19 pandemic.

	March 2020 (*n* = 814)		August 2020 (*n* = 523)		November 2020 (*n* = 250)		March 2021 (*n* = 240)		*F*	*p*	Scheffé Post hoc
	*M*	*SD*	*M*	*SD*	*M*	*SD*	*M*	*SD*			
Perceived stress	18.818	7.925	19.685	7.327	18.800	6.875	19.688	7.075	0.715	0.543	-
Worry	47.410	14.297	49.155	14.691	45.920	12.936	47.917	11.555	2.218	0.084	-
State anxiety	20.808	7.871	20.138	7.048	19.244	6.167	21.000	6.672	2.685	0.045	-
Post-traumatic symptoms	32.958	16.490	25.880	17.411	26.380	16.421	28.204	16.827	18.289	**<0.001**	T1 > T2, T3, T4
Intrusion	11.656	5.957	9.356	6.169	9.108	6.191	9.804	6.145	16.118	**<0.001**	T1 > T2, T3, T4
Avoidance	12.103	7.067	9.363	7.200	9.648	6.628	10.538	6.820	15.927	**<0.001**	T1 > T2, T3, T4
Hyperarousal	9.199	5.493	7.161	5.746	7.624	5.216	7.863	5.461	13.112	**<0.001**	T1 > T2, T3, T4
Civic moral disengagement	54.786	14.298	56.805	15.186	58.560	17.079	56.858	20.008	4.794	**0.002**	T3 > T1
Moral justification	6.964	2.663	6.859	2.643	6.788	2.800	6.954	3.171	1.223	0.300	-
Euphemistic language	7.238	2.565	8.042	2.847	7.700	2.986	7.525	3.086	10.459	**<0.001**	T2 > T1
Advantageous comparison	5.896	2.347	6.260	2.484	6.392	2.712	6.508	3.017	4.923	**0.002**	T4 > T1
Displacement of responsibility	6.173	2.378	6.319	2.557	6.836	2.576	6.408	2.750	3.557	0.014	-
Diffusion of responsibility	6.664	2.532	6.822	2.379	7.444	2.995	6.942	2.958	5.214	**<0.001**	T3 > T1, T2
Distorting consequences	5.494	2.195	5.795	2.307	6.236	2.706	6.054	2.933	7.916	**<0.001**	T1 < T3, T4
Attribution of blame	7.474	2.773	7.755	2.896	8.108	3.164	7.692	3.185	4.284	**0.005**	T3 > T1
Dehumanization	8.883	2.733	8.952	2.640	9.056	2.835	8.775	3.037	0.084	0.969	-

Note: Bold values indicate *p* within the criteria of significance (Bonferroni-adjusted *p* < 0.0125); T1, March 2020; T2, August 2020; T3, November 2020; T4, March 2021.

**Table 4:** Differences in psychological outcomes between men and women during the first year of COVID-19 pandemic in Italy.

	Men (*n* = 549)		Women (*n* = 1278)		*F*	*p*
	*M*	*SD*	*M*	*SD*		
Perceived stress	16.937	7.401	20.140	7.361	51.996	**<0.001**
Worry	43.247	12.926	49.716	13.901	54.700	**<0.001**
State anxiety	18.624	6.794	21.202	7.359	33.814	**<0.001**
Post-traumatic symptoms	25.077	16.252	31.267	17.115	36.759	**<0.001**
Intrusion	9.370	6.188	10.851	6.119	15.191	**<0.001**
Avoidance	9.031	6.737	11.527	7.149	34.024	**<0.001**
Hyperarousal	6.676	5.075	8.889	5.678	44.940	**<0.001**
Civic moral disengagement	62.117	17.305	53.590	14.437	97.746	**<0.001**
Moral justification	8.083	3.092	6.404	2.416	128.143	**<0.001**
Euphemistic language	8.662	2.982	7.100	2.577	104.708	**<0.001**
Advantageous comparison	6.457	2.977	6.016	2.321	10.305	**0.001**
Displacement of responsibility	6.952	2.954	6.072	2.252	32.026	**<0.001**
Diffusion of responsibility	7.419	2.891	6.609	2.469	29.913	**<0.001**
Distorting consequences	6.265	2.726	5.537	2.244	31.348	**<0.001**
Attribution of blame	8.482	3.033	7.321	2.808	61.373	**<0.001**
Dehumanization	9.798	2.802	8.532	2.656	65.732	**<0.001**

Note: Bold values indicate *p* within the criteria of significance (Bonferroni-adjusted *p* < 0.0125).

## Data Availability

The data presented in this study are available on request from the corresponding author. The data are not publicly available due to privacy reasons.
